# Glyphosate and phosphate treatments in soil differentially affect crop microbiomes depending on species, tissue and growth stage

**DOI:** 10.1038/s41598-025-11430-y

**Published:** 2025-07-15

**Authors:** Niina Smolander, Benjamin Fuchs, Marjo Helander, Pere Puigbò, Manu Tamminen, Kari Saikkonen, Suni Anie Mathew

**Affiliations:** 1 Department of Biology, University of Turku, 20014 Turku, Finland; 2 Biodiversity Unit, University of Turku, 20014 Turku, Finland; 3 Department of Agroecology, Aarhus University, Forsøgsvej 1, 4200 Slagelse, Denmark; 4 Department of Animal and Food Science, Autonomous University of Barcelona, 08193 Bellaterra, Barcelona, Catalonia Spain

**Keywords:** Endophytes, Bacterial communities, Agricultural treatments, Pesticides, Fertilizers, Non-target organisms, Agrochemicals, Shikimate pathway, Microbial ecology, Microbial communities

## Abstract

Glyphosate-based herbicides (GBHs) are widely used for controlling weeds by inhibiting the shikimate pathway. However, the effects of GBH on non-target organisms, such as shikimate pathway-containing microbes, are understudied. Furthermore, the complex interactions between GBH and fertilizers are difficult to predict. Hence, we experimentally investigated the effects of GBH and phosphate fertilizer on the composition of endophytic bacterial communities of potato, faba bean and oat during early and late summer using 16S rRNA gene sequencing, and on plant growth in late summer. GBH treatments significantly affected bacterial communities of early and late summer potato roots and late summer faba bean roots, while phosphate treatments significantly affected bacterial communities of late summer potato leaves, tubers and early summer faba bean leaves. The treatments reduced bacterial diversity in potato and oat and the abundance of putatively beneficial bacteria in potato and faba bean. However, these treatments increased the aboveground biomass of all crops. Thus, agrochemicals had variable effects across crops, tissues and growth stages. While improved crop yield is often prioritized in chemical-intensive farming, the effects of microbiome shifts on crop health need further investigation.

**Supplementary Information:**

The online version contains supplementary material available at 10.1038/s41598-025-11430-y.

## Introduction

Plants harbor a diverse array of microbes whose microbial constituents engage in complex interactions with each other and the host plant. The genetic constitution of these microbial partners, the ‘microbiome’, plays important roles in nutrient acquisition, disease resistance and stress tolerance for the host plant^1^. Understanding plant microbiomes has gained significant attention in agriculture and environmental research due to their potential applications in sustainable agriculture and ecosystem management^2^.

Plants provide several niches for the growth and proliferation of microbes, including the rhizosphere, phyllosphere and endosphere. A variety of factors affect the composition and dynamics of microbial communities. Plant species and genotype, plant tissue, developmental stage, phytohormones, and other plant-derived compounds and microbe-microbe interactions are examples of plant-specific and microbe-specific factors^3–6^. Moreover, environmental factors are important in shaping plant microbiomes. Natural edaphic factors and chemical-intensive agricultural practices heavily influence these microbial communities^5,7^.

Herbicides and fertilizers are often used together in agricultural practices to improve crop productivity. However, unintentional and often overlooked antimicrobial effects of these agrochemicals can deteriorate microbiomes in the environment^8^. Glyphosate-based herbicides (GBHs), one of the most widely used herbicides in the world, are broad-spectrum weed killers prevalent in agriculture, horticulture, silviculture, and urban gardening. Until recently, they have also been used in the EU to desiccate crops during pre-harvest, renew grasslands and terminate cover crops. But with the recent renewal of glyphosate use in 2023, glyphosate application is now limited to perennial weed control in arable cropping systems, conservation agriculture, and weed control in tree crops^9^. Glyphosate, the active ingredient in GBH, inhibits the EPSPS (5-enolpyruvylshikimate-3-phosphate synthase) enzyme in the shikimate pathway in plants, thus disrupting the metabolic pathway for producing essential amino acids tryptophan, tyrosine, and phenylalanine^10^. However, the presence of shikimate pathway in several microbes raises a concern about how GBH may affect non-target organisms. Microbes can be sensitive or resistant to glyphosate, depending on the type of EPSPS enzyme they have^11^. Studies have shown that glyphosate residues remain in the soil for decades, long after the 2-week degradation period claimed by manufacturers, depending on the geographical location, climate, and soil type^12,13^. There are considerable risks of glyphosate residues accumulating in the soil with each application, as many microbes are sensitive to glyphosate^11^ and thus can harm soil microbes^14^. Furthermore, the microbes in the non-target crops may be affected either via glyphosate-induced changes in the phytohormones of the host crop^14–18^ or potentially more directly via the uptake of glyphosate residues from the soil^19^.

Alongside herbicides, various fertilizers are used in agriculture to improve soil quality and increase crop yield. About 40% of the global arable land is limited by phosphorous (P) deficiency affecting crop yield, making phosphate application an essential agronomic measure. Crops only use 10–25% of the applied phosphate fertilizer, while the remaining 85% accumulates in the soil^20^. However, as glyphosate and phosphate compete for the same sorption sites, the availability of phosphate is problematic in agricultural land with the accumulation of both agrochemicals^21,22^. Furthermore, the interaction between these agrochemicals varies depending on the soil type, pH and climate^21^. Most of the research on agriculture and agrochemical interactions is focused on tropical agroecosystems owing to year-round productivity, diverse staple crops, and socio-economic factors while northern agroecosystems are less studied due to limited productivity and short growing season. Hence, agrochemical interactions in these colder ecosystems have often been overlooked. Further, most studies focus on soil or rhizosphere communities, while shifts in plant microbial communities, especially in the phyllosphere and endosphere remain elusive^23^.

Previously, we have observed that glyphosate and phosphate affect the endophytic microbial communities in strawberries and potatoes differently, depending on the host plant and tissue type^17,18^. These studies, however, focused on the shifts in microbial communities specific to each plant species, regardless of the plant’s growth stage. The impact of herbicide-fertilizer interactions on crop microbial communities is complicated, especially in multi-crop fields, across different tissue types and growth or developmental stages of the host crops. For instance, the effects of GBH separately or in combination with phosphate applications on microbes associated with early vegetative stages of the host crop could differ from the later flowering stages.

Here, we studied using a well replicated long-term field experiment, the effects of GBH (G), phosphate fertilizer (P), and GBH in combination with phosphate fertilizer (GP) on bacterial communities of potato, oats, and faba bean tissues during early growth i.e., vegetative and later mature i.e., flowering stages at the experimental field site in Southwestern Finland. These crop species were chosen for the study due to their agricultural significance in Northern Europe. The experimental study plots have been treated with recommended field-realistic doses of GBH since 2013 and phosphate since 2018, to study the effects of agrochemicals on non-target organisms, in northern ecosystems with short growing season. Agricultural applications in Europe are based on the concentration of the active substance ‘glyphosate’ found on GBH product labels and application rates depend on crop type and purpose^24^. We expect that this 10-year history of agrochemical use leading to accumulation of glyphosate and phosphate residues in soil has influenced the diversity of microbes colonizing agricultural crops. We hypothesized that the composition of bacterial communities is affected differently in aboveground and belowground tissues by each treatment at different growth stages and species of host crops. Furthermore, we assumed that changes in bacterial community compositions depend on the host plant species recruiting microbes from the soil. We collected leaf and root samples from all plants, nodules from faba bean at two time-points (vegetative and flowering stages) and tubers from potato at the flowering stage, followed by targeted 16S rRNA gene sequencing. Since plant microbiomes are crucial for plant growth, we measured the biomass of the shoot to assess if changes in bacterial communities affected plant growth. Additionally, we predicted the potential sensitivity of bacteria towards glyphosate using the novel EPSPSClass bioinformatics tool^11^ to identify if the treatments affected the abundances of glyphosate-sensitive or glyphosate-resistant bacteria.

## Results

### Taxonomic distribution of bacterial communities

A total of 9,047,850 reads were clustered into 13,263 Amplicon Sequence Variants (ASVs) belonging to 35 phyla, 192 orders, and 307 families. Proteobacteria was the dominant bacterial phylum across all crop species, tissues and growth stages. Early leaves of all crop species were characterized by Proteobacteria and Firmicutes. This trend persisted in late faba bean leaves, whereas in late growth stage leaves of potatoes and oats, the bacterial composition shifted to dominance of Proteobacteria and Bacteriodota.

In potato roots and tubers, Proteobacteria and Bacteriodota dominated both early and late stages with no significant shifts during plant development. In faba bean, Proteobacteria and Firmicutes were abundant in early roots, which shifted to Proteobacteria and Bacteriodota in the late roots. The opposite trend was observed in oat roots. In faba bean nodules, the major phylum was Proteobacteria in both growth stages (Supplementary Fig. 1).

The main bacterial orders found in different potato tissues consisted of Burkholderiales, Rhizobiales and Sphingomonadales. In the faba bean tissues, the main orders found were Burkholderiales, Rhizobiales, Bacillales, and Sphingomonadales, and in oats, they were Burkholderiales, Rhizobiales, Bacillales, and Xanthomonadales (Supplementary Fig. 2).

### Treatments reduce alpha diversity in potato leaves

**Fig. 1:**
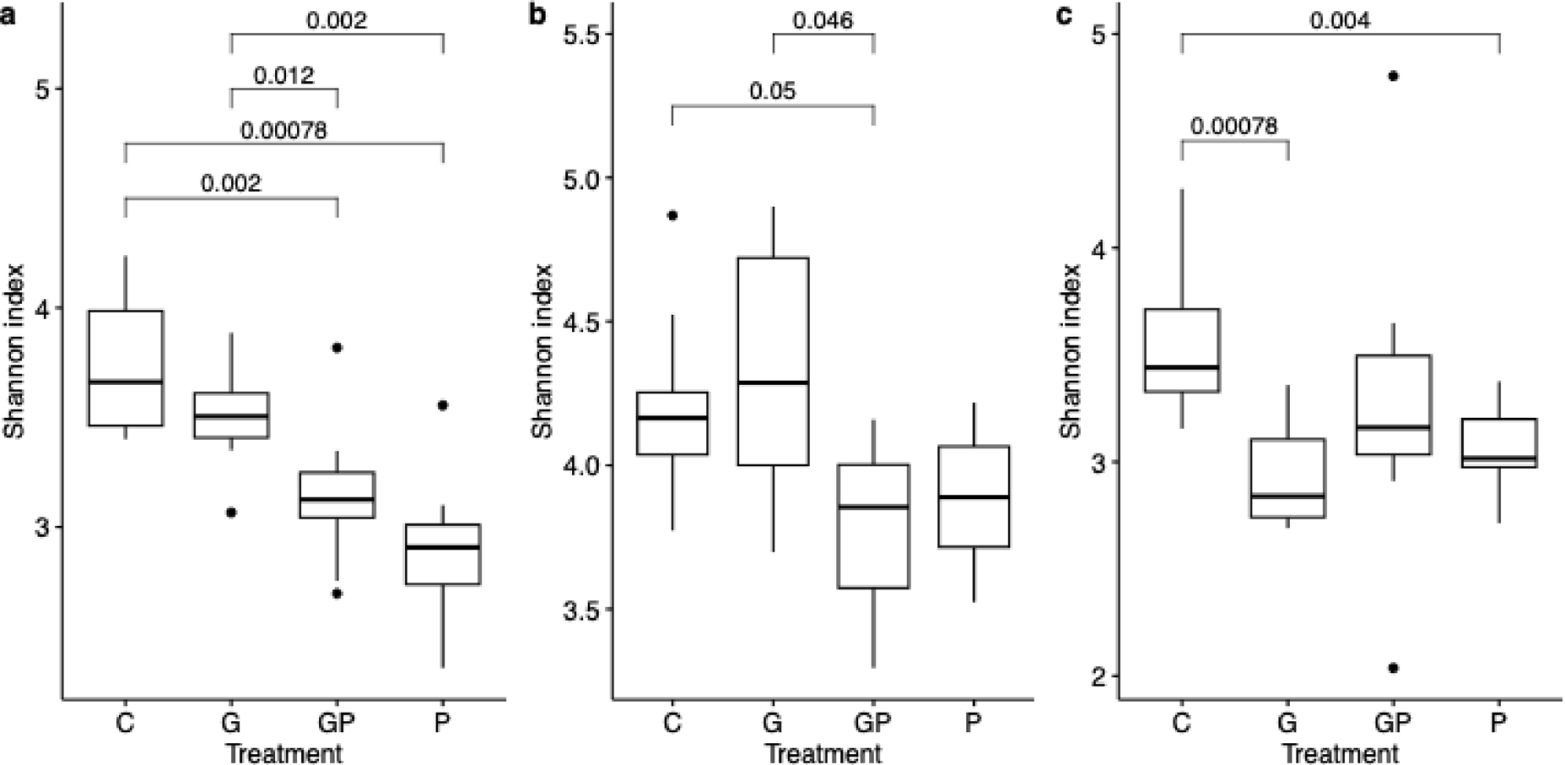
Boxplots of bacterial alpha diversity by Shannon indices. Comparing control (C), glyphosate-based herbicide (G), G with phosphate (GP) and phosphate (P) treatments in the (**a**) late growth stage potato leaves (n = 10), (**b**) late potato tubers (n = 10, except for n = 9, for P and GP) and (**c**) early oat leaves (n = 10). Significant comparisons are highlighted with Holm-adjusted *p-*values. Non-significant *p-*values not shown.

Shannon index was used to evaluate the alpha diversity of the bacterial communities in the different treatment groups in different tissues and growth stages of different crops (Supplementary Data 1). Significant differences between treatments were observed in the late growth stage potato leaves. The alpha diversity of the bacterial community was significantly lower in the GP and P treatments than in the no-phosphate groups (C, G) in the late potato leaves (Wilcoxon rank sum test, *p*-value cut-off ≤ 0.05, Fig. 1a). The alpha diversity tended to decrease in G treatments in the late potato leaves. Similarly, in potato tubers, the alpha diversity was generally lower in phosphate treatments (P and GP), with significant reduction in GP treatment (Fig. 1b). Furthermore, in the early oat leaves, G and P treatments significantly reduced the alpha diversity compared to the control (Fig. 1c). In other growth stages, crops or tissues, the differences in alpha diversity compared to the control treatment were not significant.

### GBH and phosphate shifted the composition of bacterial communities depending on plant tissue and growth stage

**Fig. 2:**
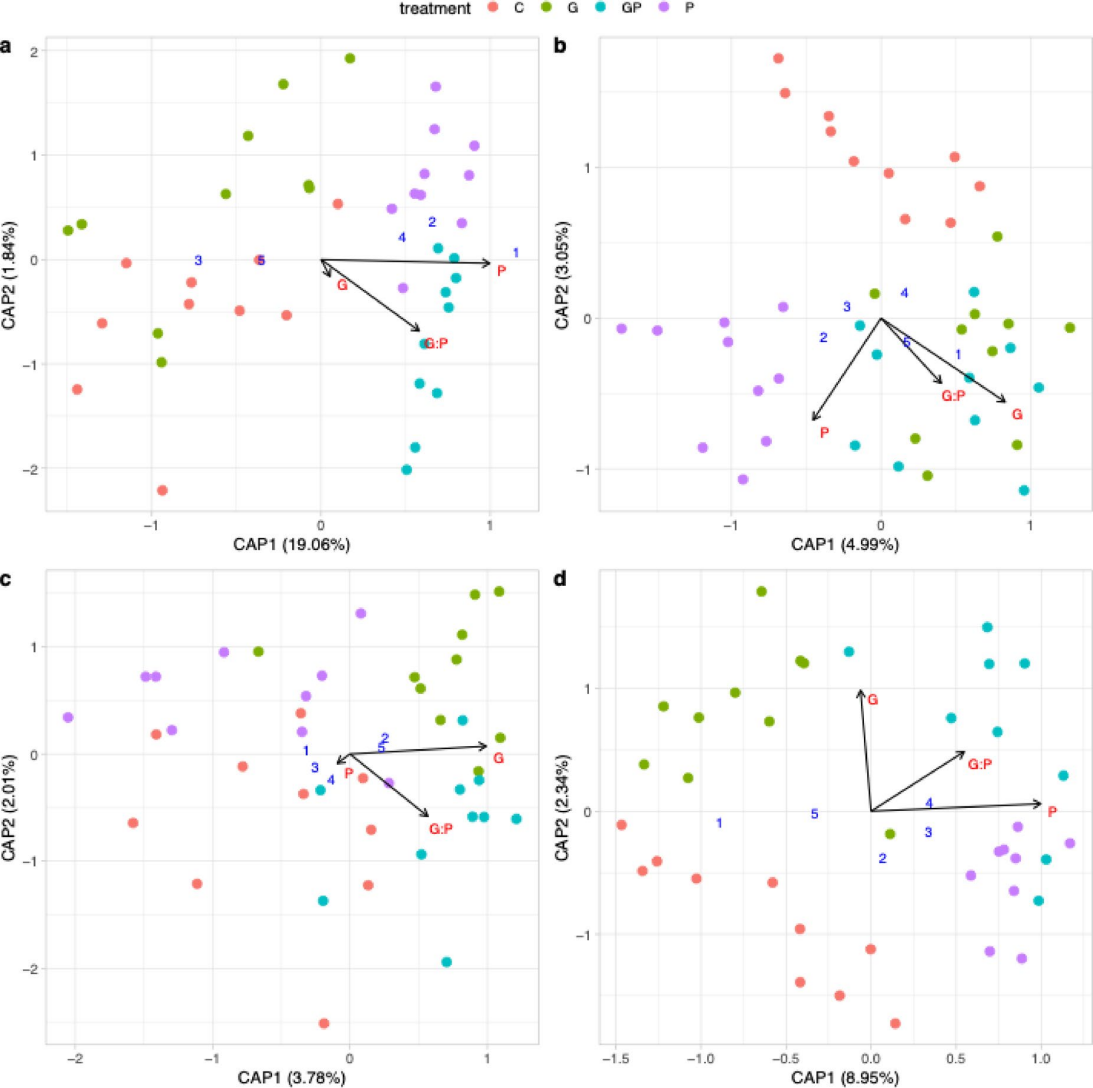
Constrained analysis of principal coordinates (CAP) ordination of bacterial communities of different potato tissues. Potato (**a**) late growth stage leaves, (**b**) early roots (**c**) late roots and (**d**) tubers. Samples are indicated as colored circles with the control (C) treatment in red, glyphosate-based herbicide (G) treatment in green, G with phosphate (GP) treatment in teal and phosphate (P) treatment in purple (n = 10 each, except for tuber GP and P n = 9 each). The most variable bacterial ASVs in response to the treatments are indicated as blue numbers (see Table 1 for details). The arrows indicate the effect of glyphosate (G) treatments, phosphate (P) treatments, and their combination (GP).

To evaluate the significance of the treatment effects on the bacterial community compositions, a constrained analysis of principal coordinates (CAP) was performed. Shifts in bacterial community compositions were observed in all potato tissues as well as in faba bean and oat leaves and roots at different growth stages (Supplementary Data 2). In potato leaves, shifts in bacterial communities were only seen in the late growth stage, where P treatments shifted bacterial communities significantly (PERMANOVA F_1,36_ = 8.78, *p* = 0.001, Fig. 2a). In potato roots, G treatments significantly shifted bacterial communities in early (PERMANOVA, F_1,36_ = 1.75, *p* = 0.002, Fig. 2b) and in late growth stages (F_1,36_ = 1.46, *p* = 0.045, Fig. 2c). Additionally, P treatments shifted the tuber communities (PERMANOVA F_1,34_ = 3.48, *p* = 0.001, Fig. 2d).

**Fig. 3:**
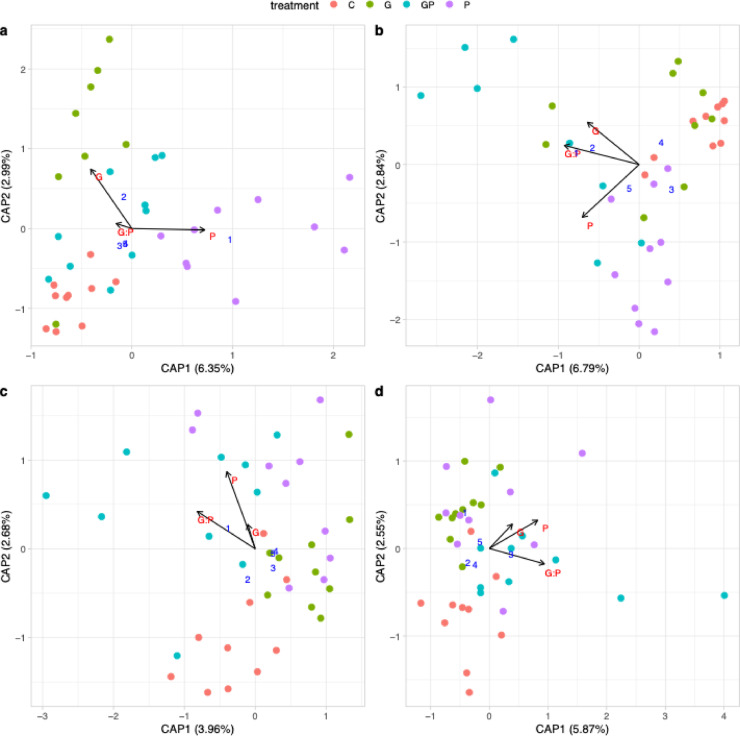
Constrained analysis of principal coordinates (CAP) ordination of bacterial communities of different faba bean and oat tissues. (**a**) Faba bean leaves in early growth stage, (**b**) faba bean roots in late growth stage, (**c**) oat leaves early growth stage and (**d**) oat roots at late growth stage. Samples are indicated as colored circles with the control (C) treatment in red, glyphosate-based herbicide (G) treatment in green, G with phosphate (GP) treatment in teal and phosphate (P) treatment in purple (n = 10 each, except for faba bean early leaf G n = 8 and root late G n = 9 and GP n = 8). The most variable bacterial ASVs in response to the treatments are indicated as blue numbers (see Table 1 for details). The arrows indicate the effect of glyphosate (G), phosphate (P), and their combination (GP).

In early growth stage faba beans leaves, P treatments and the GP treatment significantly affected the community compositions (PERMANOVA F_1,34_ = 1.83, *p* = 0.003 and F_1,34_ = 1.49, *p* = 0.029 for P and GP, respectively, Fig. 3a). In late faba bean leaves, the assumption of homogeneity of variances was not met (ANOVA *p* = 0.047), however P treatments had potentially significant effect on the community compositions (PERMANOVA F_1,36_ = 2.99, *p* = 0.002, Supplementary Data 2). Additionally, in the late growth stage roots, the shifts were significantly driven by both G and P treatments (PERMANOVA F_1,33_ = 1.66, *p* = 0.032 and F_1,33_ = 1.85, *p* = 0.016, respectively, Fig. 3b). In oat, GP treatment significantly altered the bacterial community compositions of early leaves (PERMANOVA F_1,36_ = 1.47, *p* = 0.03, Fig. 3c) and P treatments altered that of the late roots (PERMANOVA F_1,36_ = 1.85, *p* = 0.013, Fig. 3d).

### Phosphate treatments impacted the abundance of multiple taxa predominantly in leaves

**Fig. 4:**
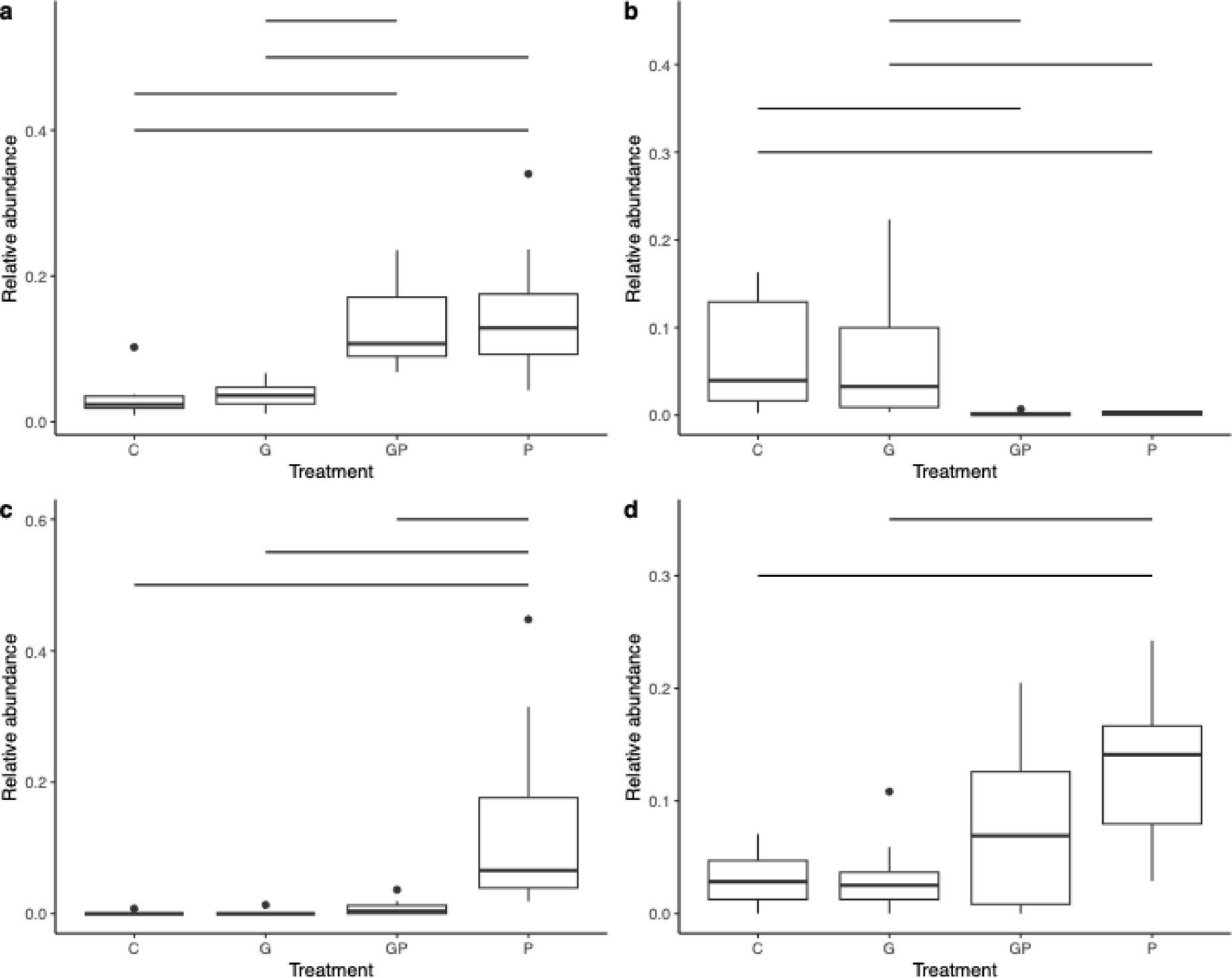
The relative abundances of the most differentially abundant bacterial ASVs. Relative abundances of (**a**) *Sphingomonas faeni* and (**b**) *Stenotrophomonas maltophilia* in late potato leaves, (**c**) *Allorhizobium-Neorhizobium-Pararhizobium-Rhizobium rhizogenes* in early faba bean leaves and (**d**) *S. maltophilia* in late faba bean leaves. Comparing control (C), glyphosate-based herbicide (G), G with phosphate (GP) and phosphate (P) treatments. Significant comparisons are defined by the minimum estimator agreement of three (out of five) and indicated with a line. The exact *p* values for each comparison and estimator are provided in the Supplementary Data 3.

To determine which bacterial abundances were significantly affected by the treatments we performed pairwise differential abundance analyses for all the plant tissues and growth stages with five different differential abundance estimators. Differentially abundant bacterial ASVs were found in late growth stages potato leaves and tubers as well as early and late growth stage faba bean leaves (Supplementary Data 3). In most of the differentially abundant ASVs, the absolute difference in abundance between treatments was low (≤ 5 percentage points, Supplementary Data 3) and major differences were only observed in three ASVs in the late potato leaves and early and late faba bean leaves (Fig. 4).

In late potato leaves, the P and GP treatments significantly increased the abundance of *Sphingomonas faeni* and reduced the abundance of *Stenotrophomonas maltophilia* compared to the C and G treatments. In early faba bean leaves, the P treatment increased the abundance of *Allorhizobium-Neorhizobium-Pararhizobium-Rhizobium rhizogenes* compared to all the other treatments. In late faba bean leaves, the P treatment significantly increased the abundance of *Stenotrophomonas maltophilia* (Table 1).

**Table 1:** The five most variable bacterial ASVs in response to treatments.

	1	2	3	4	5
Potato leaf late	*Sphingomonas faeni* ASV 9	*Hymenobacter* ASV 18	*Stenotrophomonas maltophilia* ASV 3	*Hymenobacter* ASV 60	*Delftia tsuruhatensis* ASV 6
Potato root early	*Candidatus Methylopumilus* ASV 7	*TM7a* ASV 55	*Flavobacterium* ASV 24	*Flavobacterium psychrolimnae* ASV 26	*Allorhizobium-Neorhizobium-Pararhizobium-Rhizobium* ASV 5
Potato root late	*TM7a* ASV 55	*Candidatus Methylopumilus* ASV 7	*Allorhizobium-Neorhizobium-Pararhizobium-Rhizobium* ASV 5	*Saccharimonadales* ASV 162	*Shinella* ASV 23
Potato tuber late	*Stenotrophomonas maltophilia* ASV 3	*Flavobacterium psychrolimnae* ASV 26	*Pelomonas* ASV 13	*Saccharimonadales* ASV 81	*Pseudomonas* ASV 22
Faba bean leaf early	*Allorhizobium-Neorhizobium-Pararhizobium-Rhizobium rhizogenes* ASV 2	*Pseudomonas* ASV 135	*Allorhizobium-Neorhizobium-Pararhizobium-Rhizobium* ASV 5	*Stenotrophomonas maltophilia* ASV 3	*Pantoea agglomerans* ASV 35
Faba bean root late	*Allorhizobium-Neorhizobium-Pararhizobium-Rhizobium rhizogenes* ASV 2	*Brucella melitensis* ASV 1	*Brucella* ASV 12	*Janthinobacterium* ASV 8	*Janthinobacterium* ASV 183
Oat leaf early	*Pantoea ananatis* ASV 113	*Delftia tsuruhatensis* ASV 6	*Stenotrophomonas maltophilia* ASV 3	*Bacillus halodurans* ASV 14	*Oceanobacillus profundus* ASV 21
Oat root late	*Stenotrophomonas maltophilia* ASV 3	*Delftia tsuruhatensis* ASV 6	*Pantoea agglomerans* ASV 35	*Pseudomonas* ASV 22	*Rhodanobacter* ASV 179

In potato late leaves and tubers, early and late potato roots, early faba bean and oat leaves and late roots. The column numbers refer to the blue numbers in the Figs. 2 and 3.

### Certain bacterial taxa are associated with phosphate-containing treatments in faba bean and potato during late growth stage

To identify specific bacteria taxa associated with treatments, we conducted an indicator species analysis comparing all the treatments to each other as well as compared the glyphosate-containing treatments (G, GP) to no-glyphosate (C, P) and phosphate-containing (P, GP) to no-phosphate treatments (C, G) for all the plant tissues and growth stages. In the late season potato leaves, four ASVs—ASVs 60 and 115 classified as *Hymenobacter*, ASV 124 (*Pseudomonas*) and ASV 9 (*Sphingomonas faeni)*—and ASV 124 (*Pseudomonas*) in potato tubers were associated with phosphate-containing treatments. Furthermore, 21 ASVs in late-stage potato leaves and 4 ASVs in potato tubers were associated with no-phosphate treatments: the best indicator ASVs belonging to *Stenotrophomonas maltophilia*, *Rhodococcus erythropolis,* and the genus *Allorhizobium-Neorhizobium-Pararhizobium-Rhizobium* in the late leaves and genus *Pseudomonas* and the species *Pedobacter heparinus* and *Rhodococcus erythropolis* for the tubers. In late faba bean leaves, one *Pseudomonas* ASV (ASV 607) was associated with phosphate-containing treatments, but another *Pseudomonas* ASV (ASV 124) was associated with no-phosphate treatments (Table 2).

**Table 2:** The significant bacterial indicator species and the treatments they are associated with in different plant tissues and growth stages.

Sample	Treatment	Taxa	Indicator value	*p* value
Faba bean leaf late	No-phosphate	Genus: *Pseudomonas* ASV_124	0.550	0.021
Faba bean leaf late	Phosphate	Genus: *Pseudomonas* ASV_607	0.600	0.021
Potato leaf late	No-phosphate	Species: *Stenotrophomonas maltophilia* ASV_3	0.969	0.005
Potato leaf late	No-phosphate	Genus: *Allorhizobium-Neorhizobium-Pararhizobium-Rhizobium* ASV_5	0.898	0.005
Potato leaf late	No-phosphate	Species: *Rhodococcus erythropolis* ASV_43	0.892	0.005
Potato leaf late	No-phosphate	Species: *Caldalkalibacillus thermarum* ASV_11	0.883	0.005
Potato leaf late	No-phosphate	Species: *Methylobacterium-Methylorubrum adhaesivum* ASV_160	0.860	0.005
Potato leaf late	No-phosphate	Genus: *Pseudomonas* ASV_22	0.850	0.005
Potato leaf late	No-phosphate	Genus: *Nesterenkonia* ASV_33	0.829	0.005
Potato leaf late	No-phosphate	Genus: *Halomonas* ASV_34	0.770	0.005
Potato leaf late	No-phosphate	Species: *Oceanobacillus limi* ASV_21	0.753	0.005
Potato leaf late	No-phosphate	Family: Xanthobacteraceae ASV_65	0.746	0.005
Potato leaf late	No-phosphate	Species: *Bacillus halodurans* ASV_14	0.736	0.005
Potato leaf late	No-phosphate	Genus: *Bacillus* ASV_37	0.672	0.005
Potato leaf late	No-phosphate	Species: *Bacillus halodurans* ASV_16	0.665	0.005
Potato leaf late	No-phosphate	Species: *Sphingobium yanoikuyae* ASV_143	0.650	0.005
Potato leaf late	No-phosphate	Genus: *Pseudomonas* ASV_41	0.650	0.005
Potato leaf late	No-phosphate	Species: *Methylobacterium*-*Methylorubrum adhaesivum* ASV_77	0.584	0.025
Potato leaf late	No-phosphate	Species: *Variovorax paradoxus* ASV_280	0.500	0.025
Potato leaf late	No-phosphate	Genus: *Klenkia* ASV_1381	0.500	0.012
Potato leaf late	No-phosphate	Genus: *Sphingomonas* ASV_424	0.500	0.018
Potato leaf late	No-phosphate	Family: Bacillaceae ASV_72	0.500	0.015
Potato leaf late	No-phosphate	Species: *Mesorhizobium plurifarium* ASV_158	0.500	0.015
Potato leaf late	Phosphate	Genus: *Hymenobacter* ASV_60	0.819	0.009
Potato leaf late	Phosphate	Species: *Sphingomonas faeni* ASV_9	0.803	0.005
Potato leaf late	Phosphate	Genus: *Hymenobacter* ASV_115	0.775	0.009
Potato leaf late	Phosphate	Genus: *Pseudomonas* ASV_124	0.500	0.024
Potato tuber late	No-phosphate	Genus: *Pseudomonas* ASV_22	1.000	0.043
Potato tuber Late	No-phosphate	Species: *Pedobacter heparinus* ASV_54	0.924	0.043
Potato tuber late	No-phosphate	Species: *Rhodococcus erythropolis* ASV_43	0.889	0.043
Potato tuber late	No-phosphate	Species: *Caldalkalibacillus thermarum* ASV_11	0.801	0.043
Potato tuber late	Phosphate	Genus: *Pseudomonas* ASV_124	0.611	0.043

The comparison was done between the samples grouped by the chemical: no-phosphate (C and G) versus phosphate (P and GP) and no-glyphosate (C and P) versus glyphosate (G and GP). The indicator value ranges between 0 (bad indicator) and 1 (good indicator). No significant indicator species were found for other treatment groups than phosphate and no-phosphate. In all groups n = 20, apart from Potato Tuber Late Phosphate n = 18.

### Plant tissue and growth stage can influence soil treatment effects on potential glyphosate-sensitive and potential glyphosate-resistant bacteria

**Fig. 5:**
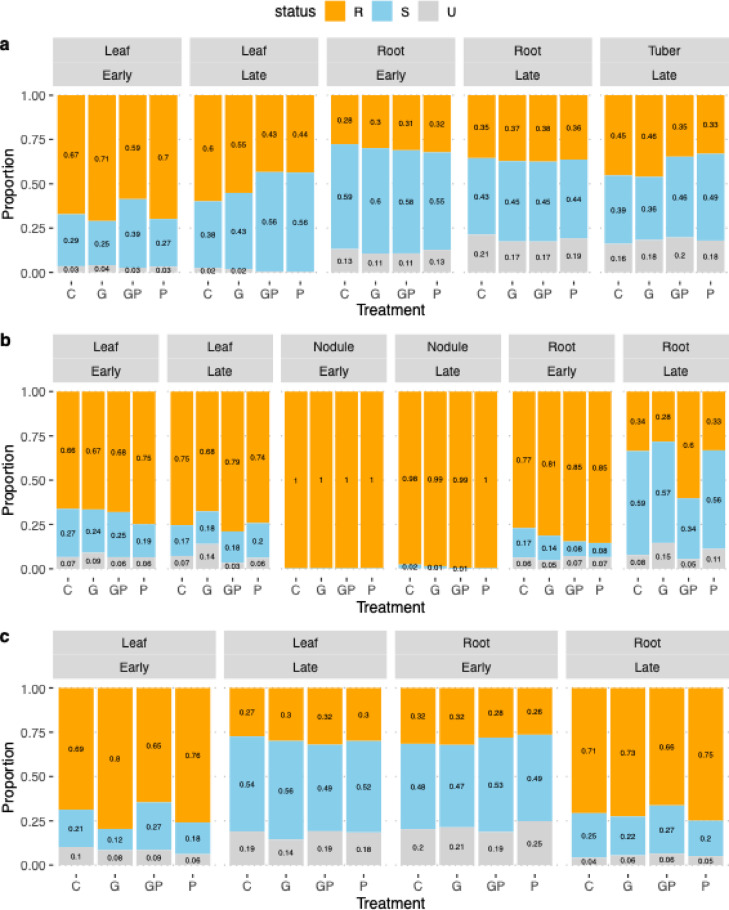
Proportions of glyphosate-sensitive (S), glyphosate-resistant (R) and unclassified (U) bacteria in all plant tissues and growth stages. Comparing control (C), Glyphosate-based herbicide (G), phosphate (P) and GBH with phosphate (GP) treatments in different plant tissues and growth stages in (**a**) potatoes, (**b**) faba beans and (**c**) oats. The values inside the bars indicate the relative abundances of sensitive, resistant and unclassified bacteria.

The potential sensitivity to glyphosate of the bacteria found in different crops, tissues, growth stages and treatments was evaluated in silico based on the taxonomic conservation of EPSPS enzyme. Overall, early growth stage potato, faba bean and oat leaves, faba bean roots and nodules as well as late faba bean leaves and nodules and oat roots had a higher relative abundance of potentially glyphosate-resistant bacteria than potentially sensitive bacteria. The opposite trend was observed in the early potato and oat roots as well as late potato roots and oat leaves (Fig. 5). The abundance of glyphosate-sensitive bacteria was significantly higher in P and GP treatment groups than in the control (C) group in the late potato leaves (Wilcoxon rank sum test, W = 17.0, *p* = 0.023 and W = 14.0, *p* = 0.016, respectively). The opposite was the case in the faba bean early leaves where phosphate reduced the abundance of sensitive bacteria compared to the control (Wilcoxon rank sum test, W = 83.0, *p* = 0.034). In early oat leaves the glyphosate treatment significantly reduced the abundance of sensitive bacteria compared to the control (Wilcoxon rank sum test, W = 86.0 *p* = 0.016).

### GBH treatments increased the biomass of aboveground tissue in all plants

**Fig. 6:**
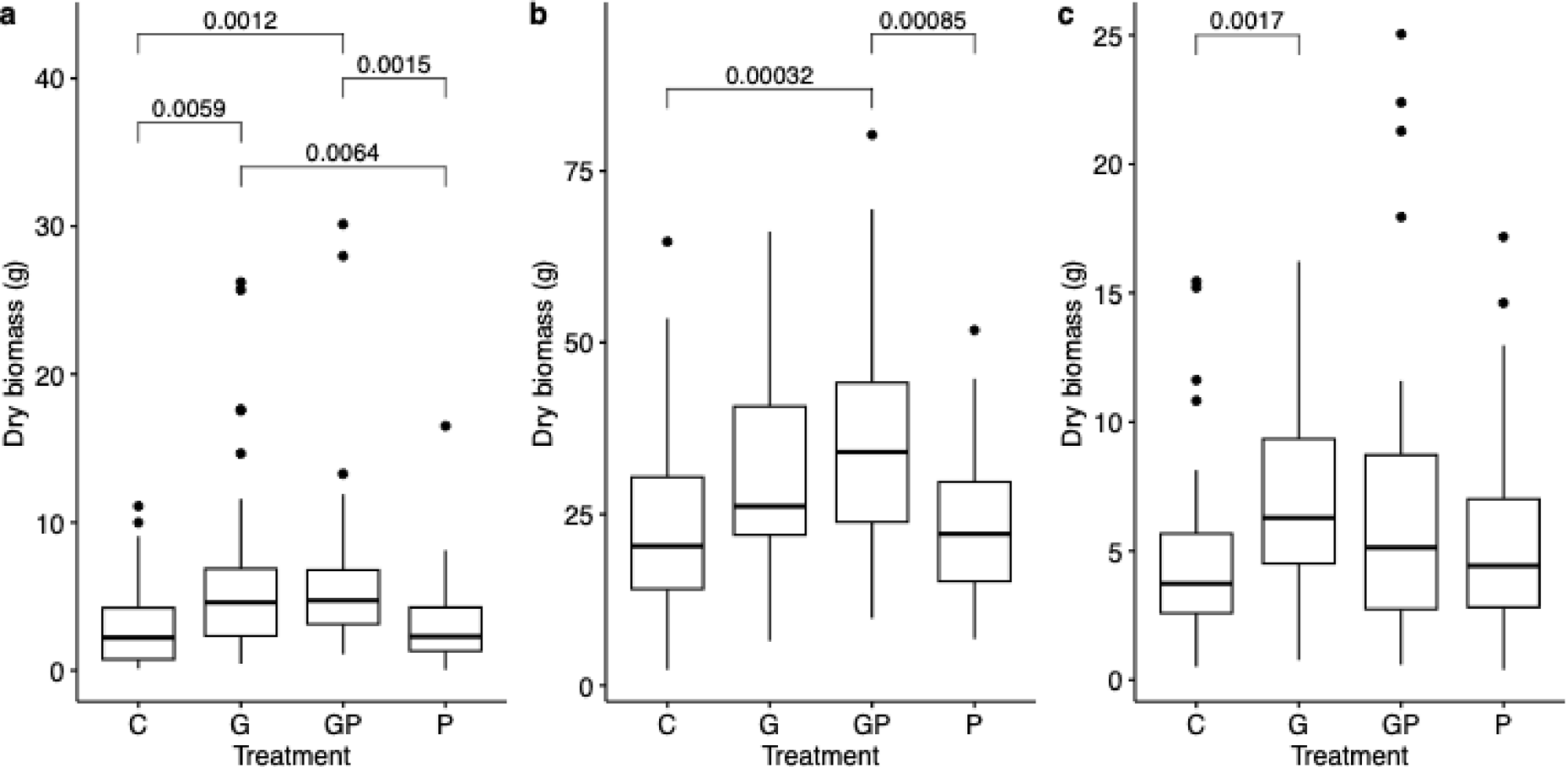
Boxplots of aboveground dry biomasses. Comparing control (C), Glyphosate-based herbicide (G), phosphate (P) and GBH with phosphate (GP) treatments in (**a**) potatoes, (**b**) faba beans and (**c**) oats. Significant comparisons are highlighted with Holm-adjusted *p*-values. Insignificant *p*-values are not shown. In all treatments n = 48, apart from in potato n = 46, n = 44, n = 36 and n = 32, for G, GP, P and C, respectively.

The effect of glyphosate and phosphate on plant biomass was evaluated by weighing the aboveground biomass of plant. Using GLM we identified that in all the plants, the effect of the G treatment was significant (t = 3.21, *p* = 0.0016; t = 2.41, *p* = 0.017 and t = 3.05, *p* = 0.0026, for potatoes, faba beans and oats, respectively, Supplementary Table 1). In potatoes, the biomass was significantly higher in the G treatment groups than in no-glyphosate groups (Wilcoxon rank sum test, *p-*value cut-off ≤ 0.05, Fig. 6). In faba beans, the plant biomass was significantly higher in the GP treatment group than the no-glyphosate groups (Wilcoxon rank sum test, *p-*value cut-off ≤ 0.05, Fig. 6). In oats, the plant biomass of the G group was significantly higher than the C group (Wilcoxon rank sum test, *p-*value cut-off ≤ 0.05, Fig. 6).

## Discussion

Our study shows that pesticide and fertilizer treatments in soil differentially affected the composition of endophytic bacterial communities in crops, mainly depending on the host species and tissue type. In addition, the effects of only G or only P and GP treatments were different in the early vegetative and late flowering stages of the crop.

Most previous studies focus on the effects of glyphosate on diversity and composition of soil and plant rhizosphere microbiomes^25–28^, overlooking plant endophytic microbiomes, especially in non-target crops. Our findings provide insight into this, as we observed glyphosate treatment-related changes in the diversity and evenness of bacterial communities as well as the abundance of potentially glyphosate-sensitive bacteria oat leaves, and changes in the community compositions in the potato and faba bean roots. Our previous studies have shown that aboveground and belowground plant tissues differ in the composition of endophytic microbial communities and that the effects of GBH and phosphate on the microbiomes are specific to the tissue type and the host plant species^15,17,18^. However, compared to our study conducted in 2019 at the same field site^18^, where G and GP treatments affected the bacterial communities in potato leaves, the two treatments had a profound effect on early growth stage potato roots, and the changes seen in leaves were driven by P and GP treatments. In both studies, P treatments significantly affected the bacterial communities in leaves and tubers. Differences in the effects of G and P treatments in leaves and roots of the two studies, which were conducted in consecutive years, could be due to variations in environmental factors, such as weather, soil pH, plant metabolites or bacterial communities in plants or soil^26^. However, both studies clearly show that the interaction between GBH and phosphate fertilizer affects the composition of endophytic bacterial communities in both aboveground and belowground potato tissues.

Bacterial community diversities and compositions varied between the early and late growth stages. In early leaves, the bacterial communities of all crops primarily contained Proteobacteria and Firmicutes, which does not change in late faba bean leaves but shifts to Proteobacteria and Bacteriodota in late potato and oat leaves. For potato roots, Proteobacteria and Bacteriodota were present in both early and late stages. However, for faba beans the early roots had Proteobacteria and Firmicutes, which shifted in the late stage to Proteobacteria and Bacteriodota. In oat roots, the trend was opposite. This aligns with findings from a study in wheat, which reported Proteobacteria, Bacteroidota, and Firmicutes as the most abundant phyla^29^. Notably, wheat leaves exhibited a higher abundance of Firmicutes, similar to our observations in early-stage oat leaves. The changes in endophytic bacterial communities in wheat were primarily influenced by plant tissue type, developmental stage, and soil nutrient availability^27^. Plant anatomy in monocots may be another influencing factor as seen in the resemblance of endophytes in wheat and oats. Plants have specific physiological needs at different developmental stages, and the metabolite niche of each tissue varies according to these physiological needs. For instance, plant root exudates recruit soil microbes depending on the need of the plant at that particular developmental or growth stage, also leading to changes in bacterial endophytic compositions^28^. Hence, we emphasize that GBH and phosphate-related effects on endophytic microbiomes depend on the host plant species, tissue type and growth stage.

The effect of GBH on the endophytic bacterial community compositions in potato roots and on the diversity in oat leaves was significantly stronger in the early summer. This could be explained by the degradation of the GBH components over time, which depends on soil properties or soil microbial communities^25,29^. Our previous study has shown that glyphosate and aminomethylphosphonic acid (AMPA) levels are higher in GBH-treated soil in late summer, 2–3 months after the treatment^17^. Higher abundance of potential glyphosate-resistant bacteria was predicted in late growth stage of oat roots. However, as the glyphosate and AMPA levels or the rate of degradation during early and late summer were not monitored in this study, the reason for the acute effects of GBH remains elusive. Changes in the bacterial community composition or diversity driven by phosphate were observed in early and late summer. In potato leaves and tubers as well as in oat roots, the changes occurred exclusively in late summer, whereas in faba bean and oat leaves, the effects were significantly stronger or limited to early summer. Some growth stage-dependent changes can be explained by the translocation of phosphate, glyphosate residues and AMPA via phloem, resulting in accumulation of the substances in leaves and removal from the roots over time^30–32^.

The phosphate-containing treatments resulted in most variations in the differential abundance analysis. Phosphate treatments (P and GP) reduced the abundance of *S. maltophilia*, a well-known P-solubilizer^33^, in late growth stage potato leaves*,* but increased its abundance in late faba bean leaves. Additionally, but to lesser extent, P treatments reduced the abundance of *R. erythropolis*, another P-solubilizer, in late potato leaves and tubers^34^. With sufficient phosphate fertilization, plants may not require these beneficial bacteria for phosphate acquisition as seen in potato. However, the two bacteria reported to be associated with potatoes are cold-adapted and may promote plant growth by providing protection against pathogens^35,36^. Therefore, although fertilization may compensate for the phosphate demands of the crop, the host immunity may be compromised, especially when these beneficial microbes are adapted for cold ecosystems. Phosphate fertilizers have varying effects on soil and plant-associated phosphate-solubilizing bacteria^34,37^, as seen for instance by the increase of *S. maltophilia* in faba bean. P and GP treatments also had varying effects on various taxa belonging to the order Rhizobiales. In potato late leaves, the abundance of the genus *Allorhizobium-Neorhizobium-Pararhizobium-Rhizobium* was relatively lower. However, in early summer faba bean leaves, the abundance of *Allorhizobium-Neorhizobium-Pararhizobium-Rhizobium rhizogenes* was relatively higher, the changes in the latter being specifically driven by phosphate. The higher abundance of *A. rhizogenes* is expected in faba beans, as in any legume, for their nodule formation and nitrogen-fixing ability^38^.

The changes in bacterial community composition and treatments may have contributed to plant biomass. Faba beans are reported to require phosphate fertilization to maintain nitrogen fixation and higher yields^39^. Enhanced phosphate fertilization from glyphosate and phosphate components from GP treatment, may have increased the aboveground biomass of faba beans. In potato and oats, the increased biomass was observed in G treatments. Our results for potatoes and faba beans are similar to our previous study^30^. The increase in biomass of aboveground tissues in G treatments could be related to the accumulation of glyphosate residues, as they can induce shikimic acid accumulation and increase photosynthesis, thus stimulating plant growth^30,40^. Low doses of glyphosate are known to enhance plant growth, an effect called hormesis. This has been reported in faba bean and mature plants of coffee^41,42^. Low doses of glyphosate promoted the growth of *Arabidopsis thaliana* under controlled conditions via modulation of root microbiome^43^. Our study shows changes in bacterial community composition, which could potentially be one of the strategies for enhanced plant growth under G treatments. Removal of weeds from the plots could also contribute to increased biomass by reducing competition for resources. However, conclusively confirming the treatment effect is challenging in field-based experiments like ours, where environmental variables are less controlled compared to laboratory settings.

Pesticide and fertilizer application guidelines are generalized and do not take into account the specific planted crops, climatic regions, cultivation methods or combinations of other chemicals. The agrochemical industries often do not disclose regulatory studies on non-target organisms, such as microbes. Our study provides valuable insights into how GBH and phosphate fertilizers significantly shift microbial communities of non-target agricultural crops. The increase in crop biomass in fertilizer treatments does not negate the potential side effects of pesticide application, which reduces the abundance of bacteria beneficial to other aspects of crop health, such as stress tolerance, metabolic activities and disease resistance. This study provides foundational information of agrochemical effects on plant microbiomes, while leaving their impact on plant health subject to further elucidation. The study paves way toward monitoring agricultural productivity that integrates crop yield and health into an overall perspective of the plant and its associated microbiome, underscoring the importance of both to be considered holistically when re-evaluating agrochemical use.

## Methods

### Experimental design

The experimental field was located at the Ruissalo Botanical Garden (60° 26′ N, 22° 10′ E), University of Turku in southwestern Finland. The soil type was medium clay with a high organic matter content (> 120 g kg^−1^) and pH 5.9 (reported previously^44^). During the experiment (June–August 2020), the average temperature was 17.1 °C, and the average precipitation was 67.4 mm.

The experimental plot has been treated with GBH Roundup Gold since 2013, and phosphate treatments were added since 2018. The GBH treatments were applied twice a year in May and October, following dosages of standard agricultural applications. The study site consisted of 20 plots (23 m × 1.5 m each) with 10 GBH-treated plots alternated with 10 control plots. Each plot was subdivided in half (two subplots in one plot), and alternating subplots were first treated with phosphate (Yara Ferticare 80 g in 10 L of water per subplot), which resulted in four treatment groups: control (C), GBH (G), phosphate (P), and GBH with phosphate (GP). The control subplots were treated with water only. Following phosphate treatments (Yara Ferticare, 80 g phosphate per plot), the GBH-treatment plots were sprayed with Roundup Gold (450 g/isopropylamine glyphosate salt, CAS: 38641-94-0, application rate: 6.4 L/ha dissolved in 3 L of tap water per plot) and the control soil plots were sprayed with the same amount of tap water (3 L per plot) (detailed description^30^) on May 20th, 2020. Weed removal was done manually from all plots. We planted 6 potato tubers (*Solanum tuberosum, var. ‘Ditta’,* organically grown), 15 oat seeds (*Avena sativa*) and 10 faba bean seeds (*Vicia faba*) on each subplot 2 weeks after the soil had been treated with GBH. Potatoes were purchased from organic farm Mikolan Luomutila, Rymättyläntie 1072, 21,130 Poikko, Finland. Oat seeds were procured from Natural Resources Institute (Luke) Finland, while faba bean were the commercially available organic seeds. These plants were chosen in the study due to their agricultural significance in Northern Europe. The same plant species have been planted annually on the field in the previous years; thus the field has the same cropping history.

### Plant tissue sampling for bacterial community analysis

Sample collections were done at two time points: early summer (July 8th & 9th, 2020) during vegetative stage (5 weeks after sowing) and late summer (August 17th & 18th, 2020) during the end of flowering stage (2 weeks before harvesting) from 10 replicate plots. Root and leaf samples were collected from one plant per treatment, so for each plant species, a total of 10 leaves and 10 root samples per replicate treatment were collected. Additionally, from each treatment, 10 faba bean nodules in early summer, 10 nodules in late summer and 10 potato tubers only in late summer were collected. After the two sampling time points, there were 200 potato samples, 160 oat samples and 240 faba bean samples, a total of 600 samples for bacterial community analysis.

About 1 g of disease-free leaves were cut from plants using sterile scissors. The plants were dug up for the root samples, and about 200–350 mg of roots were cut using sterile scissors, and for potato samples, one tuber was separated. All the samples were immediately placed into plastic bags on ice and transported to the lab. All the samples were thoroughly washed with tap water and cut into smaller pieces. Approximately 100 mg of each sample was weighed and surface sterilized in the laminar airflow hood. Samples were washed in 70% ethanol for a minute, 3% klorilli (i.e. chloramine T; Kiilto Ltd, Finland) for 3 min and then rinsed thrice in autoclaved MilliQ water for a minute each. Samples were dried, transferred to 2 ml microcentrifuge tubes and stored at − 80 °C until DNA extraction. From the third wash sample, 100 μl water was plated on Reasoner’s 2 Agar (R2A) media on Petri plate and observed for growth of bacterial colonies for a week. There was no bacterial growth, thus ensuring sterilization efficiency.

### DNA extraction and 16S rRNA gene-targeted PCR for bacterial community analysis

Frozen leaf tissue samples were homogenized (TissueLyser II Bead Mill Sample Disruption Unit, Qiagen, Germany) at maximum speed twice, for 30 s each time, and frozen root, tuber and nodule samples were homogenized twice, for 1 min each time. DNA extraction was done using Invisorb Spin Plant Mini Kit (STRATEC Biomedical AG, Germany) following the manufacturer’s instructions. DNA concentrations were checked on a Nanodrop spectrophotometer (Thermo Fisher Scientific Nanodrop ND-1000, DE, USA), diluted to 30 ng/µl for PCR template.

For bacterial community analysis, we amplified V6-V8 region of 16SrRNA gene using nested PCR approach. This approach eliminates most chloroplast 16S rRNA genes in the first PCR, resulting in a more accurate dataset by primarily amplifying bacterial genes, reducing bias, and producing shorter amplicons in the second nested PCR suited for sequencing platforms. Using primers, 799F (AACMGGATTAGATACCCKG)^45^ and 1492R (GGYTACCTTGTTACGACTT) (modified from Lane 1991^46^), we produced the first PCR amplicons. In the second PCR, we produced 350-450 bp—sized amplicons using 1:10 dilutions of the first-PCR amplicons as template along with the primer combination M13-tagged 1062F (ACGACGTTGTAAAAGTCAGCTCGTGYYGTGA^47^) and M13-tagged 1390R (CATTAAGTTCCCATTAACGGGCGGTGTGTRCAA^48^). The M13 tags help in barcoding amplicons in the third PCR where 1:1 diluted second-PCR products were templates, and the amplicons were tagged with barcodes and P1 adapter sequences for Ion Torrent Sequencing^49^. Along with the templates and primers mentioned above, PCR reactions consisted of 1 × PCR buffer, 0.2 mM dNTPs, 0.3 μM of each primer and 2000 U/ml GoTaq DNA Polymerase (Promega, WI, USA) in a 30 μl reaction volume. The amplification profile was 3 min initial denaturation at 95 °C followed by 35 cycles of denaturing at 95 °C for 45 s, annealing at 54 °C for 45 s, and extension at 72 °C for 1 min. The final extension was carried out at 72 °C for 5 min. The same profile was followed for all three PCRs except for the numbers of cycles being 25 and 8 for the second and third rounds. All PCR reactions had negative controls, and all amplicons were run on 1.5% agarose gel to ensure the presence of amplicons.

### Library preparation and sequencing

PCR amplicons were analyzed with Agilent 2100 Bioanalyzer system, and sequencing libraries were prepared. Sequencing libraries were size-fractionated on Pippin Prep (Sage Science, MA, USA) using a 2% Agarose gel cassette (Marker B) and selecting 350-500 bp amplicons; they were sequenced on Ion 316™ Chip v2 in an Ion Personal Genome Machine (PGM™) (ThermoFisher Scientific, USA).

### Plant biomass

Plant biomass of aboveground plant material was collected on the 1st and 2nd of September 2020 by cutting all remaining plants at ground level with a hand-held sickle. Plants were counted and bagged into paper bags. Plants were pooled into one paper bag for each plant species and subplot; 5–10 plants per species per subplot. All paper bags were placed in a drying room (30 °C and dehumidified air) for 7 days to ensure dry weight measurements. Subsequently, plant weight was determined on a scale (Mettler Toledo AX 204). Weight was divided by plant number per bag to calculate the average weight per plant per subplot.

### Bioinformatics and statistical analyses

The 16S rRNA gene amplicon sequencing files were processed using Nextflow v.22.04.5 pipeline Ampliseq v.2.4.0^50^ on the computing cluster Puhti provided by CSC—IT Center for Science, Finland (https://csc.fi/en/). In the pipeline, the adapter trimming was performed using Cutadapt^51^, and the raw read quality control was done using FastQC^52^. DADA2^53^ and Barrnap^54^ were used to process the files into ASVs. The taxonomical classification was performed by DADA2 with the SILVA database^55^ as the reference. Data originating from multiple sequencing runs was taken into account in the pipeline.

The statistical data analysis was performed using R v.4.4.3 and Rstudio^56,57^. Before the analysis, the ASVs belonging to the domain Eukaryota or the phylum Cyanobacteria were removed from the data. The Shannon alpha diversity indices were calculated using R package mia v.1.14.0^58^ method “addAlpha” using 100 rarefaction rounds. The rarefaction depth was determined separately for each plant tissue and growth stage sample set and was the lowest read number that was minimum 15% of the median read number (Supplementary Data 4). The significance of the differences between treatments was analyzed using two-sided Wilcoxon rank-sum test with holm multiple comparison correction from the packages ggpubr v.0.6.0^59^ and rstatix v.0.7.2^60^. Levene’s test from the R package car v.3.1–2^61^ was used to assess the assumption of equal treatment group variances and the assumption was met in all the comparisons. The mean Bray–Curtis dissimilarity between treatments was calculated using the package vegan v.2.6–4^62^ method “avgdist” at ASV-level. The rarefaction depth was determined the same way as with the alpha diversity with 100 rarefaction rounds. The constrained analysis of principal coordinates (CAP) was then performed using the vegan method “capscale” with glyphosate (G), phosphate (P) and their combination as the explanatory factors and the mean Bray–Curtis dissimilarity indices as the response variable. The significance of the results was tested using ANOVA-like permutation test with 999 permutations. The multivariate homogeneity of group dispersions of the dissimilarity indices was checked using the vegan package method “betadisper” and the assumption of multivariate homogeneity of group dispersions was met, unless otherwise stated.

Prior to the differential abundance analysis, indicator species analysis, and the EPSPS enzyme in silico analysis, the samples were rarefied using the package mia with the rarefaction depth determined separately for each plant tissue and growth stage sample set and was the lowest read number that was minimum 15% of the median read number of the set (Supplementary Data 4).

Differential abundance analysis between treatments was conducted separately for each plant tissue and growth stage at ASV-level. The taxa were filtered at the prevalence of 0.1. For the analysis, five R packages were used: ALDEx2 v.1.38.0^63^, ANCOMBC v.2.8.1^64^, GuniFrac (ZicoSeq) v.1.8^65^, dacomp v.1.26^66^ and eBay v.0.1^67^. The *p*-values were corrected using the default correction methods of the packages, except for the ANCOMBC, where the correction method was set to Benjamini–Hochberg correction to match the rest of the packages.

Dufrene-Legendre Indicator Species Analysis for different treatment groups was performed using the R package labdsv v.2.1–0^68^. For the analyses species relative abundance at the lowest available taxonomical level were used. The *p*-values were adjusted using the Benjamini–Hochberg correction.

The potential sensitivity to glyphosate of the bacteria found in all the plants, plant tissues and growth stages was evaluated using an EPSPS enzyme in silico analysis, in which bacterial ASVs at genus or higher level were mapped onto EPSPS sequences in the ATGC database^69^, as described in Mathew et al.^70^. The score of potential sensitivity to glyphosate of the taxonomic group ranges from 0 (resistant: none of the sequences in the group are putatively sensitive) to 1 (sensitive: all known sequences in the group are sensitive). Cut-off scores of < 0.2 and > 0.8 were used for resistant and sensitive, respectively. The significance of the differences between the control and other treatments was analyzed using multiple pair-wise two-sided Wilcoxon rank-sum tests with holm multiple comparison correction from the package rstatix v.0.7.2^60^. Levene’s test from the R package car v.3.1–3^61^ was used to assess the assumption of equal treatment group variances and the assumption was met in all of the comparisons.

The statistical analysis of the plant weights was performed using the R’s stats v.4.2.3 package generalized linear models (GLMs) with the plant weight as the response variable and the glyphosate (G), phosphate (P) and their combination as the explanatory factors (Gamma family, log link function). The treatments were compared using two-sided Wilcoxon rank-sum test with holm multiple comparison correction from the package ggpubr v.0.6.0. Levene’s test from the R package car v.3.1–3^61^ was used to assess the assumption of equal treatment group variances and the assumption was met in all the comparisons.

The results were visualised using ggpubr v.0.6.0 and ggplot2 v.3.5.0^71^ packages.

## Electronic supplementary material

Below is the link to the electronic supplementary material.

Supplementary Material 1

Supplementary Material 2

Supplementary Material 3

Supplementary Material 4

Supplementary Material 5

Supplementary Material 6

## Data Availability

The sequencing data is available at NCBI Sequence Read Archive database under the accession PRJNA1167921 (https://www.ncbi.nlm.nih.gov/bioproject/1167921).
