# Supplementary material for: Glyphosate and phosphate treatments in soil differentially affect crop microbiomes depending on species, tissue and growth stage
Source: Sci Rep. 2025 Jul 15;15:25502. doi: 10.1038/s41598-025-11430-y (PMC12263844; doi:10.1038/s41598-025-11430-y)
Supplement: Supplementary file 4 — Supplementary Material 4 [file 41598_2025_11430_MOESM4_ESM.docx]

Description of Additional Supplementary Files

**Supplementary Data 1:** Shannon diversity index for all the samples and Wilcoxon rank sum test results comparing Shannon diversity indices of treatments in all plant tissues and growth stages.

**Supplementary Data 2:** ANOVA-like permutation test results for the constrained analysis of principal coordinates (CAP) of bacterial communities of all plant tissues and growth stages, and *p* values for the analysis of multivariate homogeneity of group dispersions (betadisper *p* values).

**Supplementary Data 3:** Differentially abundant taxa (corrected *p*-value ≤ 0.05) and their mean abundances (%) in different treatment group comparisons in different plants, tissues and growth stages. “Mean abundance first (%)” and “n first” columns refer to first treatment comparison term in the Comparison column, the “Mean abundance second (%)” and “n second” columns refer to the second treatment. Only taxa with minimum DAA estimator agreement of 3 are shown. The table includes individual *p* values for each comparison and estimator package.

**Supplementary Data 4:** Mean, median, min, max and standard deviation of the raw and Nextflow pipeline Ampliseq filtered read numbers, as well as the rarefaction depth used in the statistical analyses and number of samples prior and post rarefaction.
