# Supplementary material for: Glyphosate and phosphate treatments in soil differentially affect crop microbiomes depending on species, tissue and growth stage
Source: Sci Rep. 2025 Jul 15;15:25502. doi: 10.1038/s41598-025-11430-y (PMC12263844; doi:10.1038/s41598-025-11430-y)
Supplement: Supplementary file 6 — Supplementary Material 6 [file 41598_2025_11430_MOESM6_ESM.docx]

Supplementary Information for

**Glyphosate and phosphate treatments in soil differentially affect plant microbiomes depending on species, tissue and growth stage of hostcrops**

Niina Smolander^1^, Benjamin Fuchs^2,3^, Marjo Helander^1^, Pere Puigbò^1,4,5^, Manu Tamminen^1^, Kari Saikkonen^2^, Suni Anie Mathew^1,2*^

1 Department of Biology, University of Turku, 20014 Turku, Finland

2 Biodiversity Unit, University of Turku, 20014 Turku, Finland

3 Department of Agroecology, Aarhus University, Forsøgsvej 1, 4200 Slagelse, Denmark

4 Eurecat, Technology Center of Catalonia, Nutrition and Health Unit, Reus, 43204, Catalonia, Spain

5 Department of Biochemistry and Biotechnology, University Rovira i Virgili, 43007 Tarragona,Catalonia, Spain

*Corresponding author: Suni Anie Mathew; suni.mathew@utu.fi

 **Supplementary Figure 1. Phylum-level taxonomic distributions of endophytic bacterial communities.** Comparing control (C), glyphosate-based herbicide (G), phosphate (P), and glyphosate-based herbicide with phosphate (GP) treatment groups in (a) potatoes, (b) faba beans and (c) oats, in different plant tissues and growth stages. ASVs outside the 14 most abundant phyla and those that could not be assigned taxonomy at the phyla level are included as "Other".

**Supplementary Figure 2. Order-level taxonomic distributions of endophytic bacterial communities.** Comparing control (C), glyphosate-based herbicide (G), phosphate (P), and glyphosate-based herbicide with phosphate (GP) treatment groups in (a) potatoes, (b) faba beans and (c) oats, in different plant tissues and growth stages. ASVs outside the 14 most abundant orders and those that could not be assigned taxonomy at the order level are included as "Other".

**Supplementary Table 1.** GLM coefficient values for potato, faba bean and oat aboveground dry biomasses.

| **Potato** | | | | |
| --- | --- | --- | --- | --- |
|  | Estimate | Std.Error | t value | Pr(>\|t\|) |
| (Intercept) | 1.09339 | 0.17042 | 6.416 | 1.64e-09 |
| glyphosateTRUE | 0.71301 | 0.22191 | 3.213 | 0.0016 |
| phosphateTRUE | 0.08381 | 0.23422 | 0.358 | 0.721 |
| glyphosateTRUE:phosphateTRUE | -0.0752 | 0.31013 | -0.242 | 0.8087 |
| **Faba bean** | | | | |
|  | Estimate | Std.Error | t value | Pr(>\|t\|) |
| (Intercept) | 3.12466 | 0.07079 | 44.143 | <2e-16 |
| glyphosateTRUE | 0.24099 | 0.10011 | 2.407 | 0.017 |
| phosphateTRUE | 0.0254 | 0.10011 | 0.254 | 0.8 |
| glyphosateTRUE:phosphateTRUE | 0.15815 | 0.14157 | 1.117 | 0.265 |
| **Oat** | | | | |
|  | Estimate | Std.Error | t value | Pr(>\|t\|) |
| (Intercept) | 1.5179 | 0.102 | 14.876 | <2e-16 |
| glyphosateTRUE | 0.4401 | 0.1443 | 3.05 | 0.00262 |
| phosphateTRUE | 0.1762 | 0.1443 | 1.221 | 0.22353 |
| glyphosateTRUE:phosphateTRUE | -0.2367 | 0.2041 | -1.16 | 0.24754 |
